# Effect of pectin, lecithin, and antacid feed supplements (Egusin^®^) on gastric ulcer scores, gastric fluid pH and blood gas values in horses

**DOI:** 10.1186/1746-6148-10-S1-S4

**Published:** 2014-07-07

**Authors:** Michelle C Woodward, Nan K Huff, Frank Garza, Michael L Keowen, Michael T Kearney, Frank M Andrews

**Affiliations:** 1 Veterinary Clinical Sciences, School of Veterinary Medicine, Louisiana State University, Baton Rouge, LA, 70803, USA; 2 Pathobiological Sciences, School of Veterinary Medicine, Louisiana State University, Baton Rouge, LA. 70803, USA

**Keywords:** horse, stomach, gastric ulcers, pH, pectin, lecithin, calcium carbonate, sodium bicarbonate

## Abstract

**Background:**

The objectives of this study were to evaluate the effects of two commercial feed supplements, Egusin 250^®^ [E-250] and Egusin SLH^®^ [E-SLH], on gastric ulcer scores, gastric fluid pH, and blood gas values in stall-confined horses undergoing feed-deprivation.

**Methods:**

Nine Thoroughbred horses were used in a three-period crossover study. For the three treatment groups, sweet feed was mixed with E-250, E-SLH, or nothing (control group) and fed twice daily. Horses were treated for 21 days, then an additional 7 days while on an alternating feed-deprivation model to induce or worsen ulcers (period one). In periods two and three, horses (n=6) were treated for an additional 7 days after feed-deprivation. Gastroscopies were performed on day -1 (n=9), day 21 (n=9), day 28 (n=9) and day 35 (n=6). Gastric juice pH was measured and gastric ulcer scores were assigned. Venous blood gas values were also measured.

**Results:**

Gastric ulcers in control horses significantly decreased after 21 days, but there was no difference in ulcer scores when compared to the Egusin® treated horses. NG gastric ulcer scores significantly increased in E-250 and control horses on day 28 compared to day 21 as a result of intermittent feed-deprivation, but no treatment effect was observed. NG ulcer scores remained high in the control group but significantly decreased in the E-SLH- and E-250-treated horses by day 35. Gastric juice pH values were low and variable and no treatment effect was observed. Mean blood pCO_2_ values were significantly increased two hours after feeding in treated horses compared to controls, whereas mean blood TCO_2_ values increased in the 24 hour sample, but did not exceed 38 mmol/l.

**Conclusions:**

The feed-deprivation model increased NG gastric ulcer severity in the horses. However, by day 35, Egusin^®^ treated horses had less severe NG gastric ulcers compared to untreated control horses. After 35 days, Egusin^®^ products tested here ameliorate the severity of gastric ulcers in stall-confined horses after feed stress.

## Background

Nonglandular (NG) gastric ulcers, as part of the equine gastric ulcer syndrome (EGUS), are common in performance horses, and the current FDA-approved pharmaceutical agent in the United States, omeprazole (GastroGard® paste, Merial Limited, Decatur, GA, USA), is effective in treatment [1-16]. However, pharmaceutical agents are expensive, require a prescription, must be administered orally, and gastric ulcer recurrence is common once treatment is discontinued [15-17]. Development of a less expensive and natural alternative that could be added to the feed would be desirable.

Recently, there has been increased interest in the use of feed supplements to treat and prevent gastric ulcers in horses, and many commercial products are available [18-20]; however, little data exists on the efficacy of these supplements. Two such commercially available feed supplements containing dried apple pectin pulp and other fruit products, lecithin, sodium bicarbonate, calcium bicarbonate, dehydrated alfalfa meal, insoluble oat fiber (ß-glucan), polar lipids, and natural antioxidants (Egusin 250^®^ (E-250) and Egusin SLH^®^ (E-SLH), Centaur Animal Health, Overland Park, KS, USA) are commercially available as aids in promoting a healthy stomach and as aids in preventing the destruction of the stomach lining (manufacturer claim) [21]. Several ingredients in these feed supplements been shown in other species to support and protect the stomach lining, support normal digestive function, bind bile acids, and protect the stomach against the generation of damaging oxygen free radicals [19,20,22-26]. In addition, antacids like calcium carbonate have been shown to protect the nonglandular (NG) mucosa *in vitro* from the damaging effects gastric acids [27,28]; however, there are no critical studies on the use of this commercial supplements in horses.

The purpose of this study was to evaluate the effect of E-SLH powder and E-250 pellets on NG gastric ulcer scores, gastric juice pH, and blood gas values in stall-confined horses undergoing feed deprivation. We hypothesize that E-SLH- and E-250-treated horses will have less severe NG gastric ulcers, higher gastric juice pH, and similar blood gas values compared to untreated control horses.

## Methods

All procedures were approved by the Louisiana State University (LSU) Institutional Animal Care and Use Committee (LSU IACUC Protocol #10-078). All horses used in the study were Thoroughbred or Thoroughbred-cross breeds (n=9; 4-11 years of age, 5 geldings & 4 mares, 429-593 kg body weight) from the resident Equine Health Studies Program (EHSP) herd at LSU School of Veterinary Medicine. The study was performed in the fall and winter, from September 23, 2010 through February 15, 2011. Before the beginning of the study, a physical examination was performed on all horses in order to exclude presence of clinical disease. All horses were vaccinated in the spring for Eastern Equine Encephalitis, Western Equine Encephalitis, West Nile Virus Encephalitis, Tetanus, and Influenza and dewormed with a product containing ivermectin or moxidectin (200 μg/kg body weight, orally, twice yearly) as part of the routine herd care. For period 1 of the study, horses enrolled were stratified by equine gastric ulcer syndrome (EGUS) score [15] as recorded on day -1, after gastroscopy examination, and then randomly assigned to one of three treatment groups (three horses per treatment group) by drawing numbers out of a hat: control (untreated), E-250 (125 gm, top-dressed on sweet feed, twice daily) and E-SLH (50 gm, top-dressed on sweet feed, twice daily). For period 2, horses were randomly assigned to the alternate two treatment groups (three horses per treatment group) by drawing a number out of a hat. For period 3, horses were assigned to the treatment groups (three horses per treatment group) that they had not been assigned to previously. The treatment groups were equal (n=3) for all the treatment periods, including periods 2 and 3.

The experiment was performed as a three-period, three-treatment crossover design. Horses were housed in stalls and fed sweet feed (1 kg, twice daily, Omolene 100, Purina Mills, St. Louis, MO, USA). Horses were also fed mixed grass hay (1.5% of body weight) divided into two equal feedings per day. Horses served as their own controls.

The day prior to the beginning of each period (Day-1), horses were taken out of the pasture and housed in non-environmentally controlled 3 m x 3 m stalls. Horses in the treatment groups received E-250 or E-SLH top-dressed on sweet feed twice daily during treatment periods, while control horses received the same amount of sweet feed without supplement added. At the end of 21 days of treatment, horses were subjected to a modified intermittent feed-deprivation protocol to cause or worsen existing NG gastric ulcers [29]. Briefly, during the feed-deprivation periods, horses were muzzled and deprived of feed for 24 hours, then fed their normal ration for 24 hours until a total of 96 hours of cumulative feed-deprivation was achieved. Horses were muzzled and wood-shavings were removed from the stalls during the feed-deprivation period to prevent ingestion of bedding and manure. E-250 and E-SLH-treated horses continued to receive treatment twice daily top-dressed on sweet feed, while control horses received the same amount of sweet feed without treatment. After each period, horses were turned out in the pasture, where they were maintained on native grass pastures supplemented with native round-bale grass hay for at least 2 weeks to prevent carry-over treatment effects. Period 1 was 28 days in length and periods 2 and 3 were 35 days in length. Periods 2 and 3 were lengthened to determine the effect of treatment on recovery from the feed-deprivation model, and each treatment group continued to have three horses. In addition, a longer treatment for periods 2 and 3 was added, as a previous study in horses treated with pectin and lecithin did not show a treatment effect until after being treated for 30 days [20]. Horses had free access to water at all times. After period 3 horses were returned to the EHSP herd.

Gastroscopy was performed on all horses on days -1, 21, and 28 of treatment in period 1, and on days -1, 21, 28, and 35 of treatment in periods 2 and 3. Gastroscopic examination was performed using a 3-m endoscope (Karl Storz, El Segundo, CA, USA). To improve visualization of the stomach, feed was withheld for 16-18 hours and water for 3-4 hours prior to gastroscopy. Horses were sedated with xylazine (0.4 mg/kg, IV; AnaSed^®^, Lloyd, Shenandoah, IA, USA) prior to gastroscopic examination. To enable observation of the NG squamous mucosa (fundus ventriculi), margo plicatus, and glandular mucosa (corpus ventriculi), the stomach was insufflated with air using an air compressor (Airhead® High Pressure Air Pump, Kwik Tek, Denver, CO, USA) attached to the endoscope biopsy channel until the rugae, or stomach folds, disappeared. The mucosa was rinsed of adherent food material and mucus with tap water flushed through the endoscope biopsy channel. Each horse’s stomach was assigned an EGUS gastric ulcer score by one of the authors (FMA) masked to treatment groups, based on the EGUS ulcer scoring system where, score 0 = Intact mucosa (can have reddening and/or hyperkeratosis), score 1 = small single or small multifocal ulcers, score 2 is large single or large multifocal ulcers, and score 3 = extensive (often coalescing) ulcers with areas of apparent deep ulceration [15]. In addition, glandular ulcers were counted and recorded.

Gastric fluid was aspirated through the biopsy channel of the endoscope prior to insufflation of the stomach or addition of tap water and pH was measured within 30 minutes using a benchtop pH meter (ThermoOrion pH Meter Model 410A, Thermo Fisher Scientific, Beverly, MA).

Venous blood samples were collected from the jugular vein in heparinized blood gas syringes (Arterial Blood Sampler, Radiometer Medical ApS, Brøshøj, Denmark), air bubbles were expressed, the needle removed and syringe capped, and immediately placed in ice. The blood gas samples were obtained from all horses on the first three days of treatment Periods 1 and 3. Samples were collected prior to treatment and 2, 6, 12 (before the second treatment), 24 (before the third treatment), and 48 hours (before the fourth treatment) after treatment. These samples were analyzed using an automated blood gas analyzer (Rapidpoint 405, Siemens, Norwood, MA) for bicarbonate concentration ([HCO_3_]), total carbon dioxide (TCO_2_) and partial pressure of carbon dioxide (pCO_2_).

Data were pooled and means and standard error of the means (SEM) were reported on all variables using the 35-day values taken from horses completing periods 2 and 3. Data were analyzed statistically using a statistical analysis package for personal computers (SAS® 9.1, SAS Institute, Inc., Cary, NC). A repeated measures analysis of variance (ANOVA) using a mixed effects model (proc MIXED) was used to analyze the data. Fixed effects in the model included Treatment, Period and the interaction of Treatment*Period. When significant differences were found, post-hoc pairwise comparisons were conducted with t-tests of least-squares means for main effects and for interaction effects. Significant was set at P < 0.05.

## Results

Physical examinations were within normal limits and there was no evidence of clinical gastrointestinal disease in any of the horses prior to enrollment in the study. One horse entered the trial with a laceration over the metacarpal III bone. This wound was routinely cleaned and bandaged throughout the course of the study and no medication was administered for the wound. Feed quantities (hay) were adjusted so that body weight did not change significantly during the trial. The amount of hay required to maintain body weight was not evaluated statistically.

E-250, a pelleted formulation, when mixed with sweet feed, was readily consumed by all horses. E-SLH, a powder formulation, when mixed with sweet feed, was consumed by all horses, but some horses took several hours to consume the product and the powder immediately sifted to the bottom of the grain bucket when top-dressed.

One untreated horse and one E-250-treated horse developed abdominal pain (colic) during period 1 and the final day of period 3 of the study, respectively. In both of these horses, clinical signs resolved with flunixin meglumine (1.1 mg/kg, IV, once) and mineral oil (2 liters, via nasogastric tube) administration.

A period effect was not noted.

### Gastric ulcer scores

**Figure 1:**
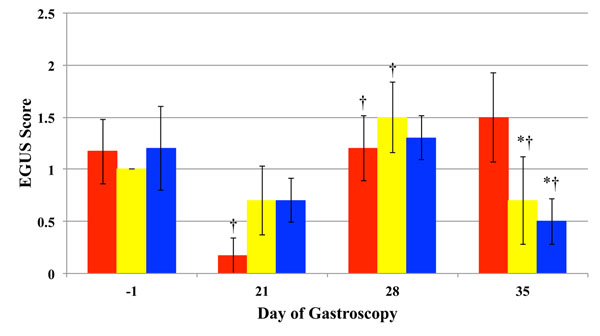
**Gastric ulcer scores** Mean ± SEM of Equine Gastric Ulcer Syndrome (EGUS) scores in horses fed Egusin 250^®^ Supplement, Egusin SLH^®^ Supplement, and controls during periods two and three before treatment (Day -1), after 21 days of treatment (Day 21), after 28 days of treatment (Day 28), and after 35 days of treatment (Day 35). A 7-day alternating feed-deprivation protocol was employed between Days 21 and 28. † Denotes a significant (*P* < 0.05) difference from scores from the previous week within a group and * denotes significant differences (P < 0.05) from control values for the same week. (controls = red bar; E-250 = yellow bar; E-SLH = blue bar)

There was no significant difference in mean EGUS scores between treatment groups on day 1, prior to beginning the study. However, mean EGUS scores decreased significantly (p<0.05) in the control group after 21 days of treatment, when compared to day 1, but there was no difference in ulcer scores when compared to the Egusin^®^ treated horses on day 21. On day 28 of treatment, mean EGUS scores significantly increased in the control and E-250 groups, indicating that the feed-deprivation model was effective in inducing or worsening gastric ulcers. In addition, mean gastric ulcer scores did not significantly increase in the Egusin SLH^®^-treated horses at day 28 when compared to day 21. However, there was no treatment effect at this time point. By day 35 of treatment, one week after the feed-deprivation period, mean EGUS scores significantly decreased in the E-SLH- and E-250-treated horses, when compared to untreated controls at the same time point and scores on day 28 of the same treatment group. Also, by day 35 of treatment, individual EGUS scores either decreased or stayed the same in all Egusin^®^-treated horses (Figure 1).

### Glandular ulcers

Glandular ulcers were observed in 6/72 (8%) gastroscopic examinations performed in this study. There was no significant day or day by treatment effect on the number of glandular ulcers in this study.

### Gastric fluid pH

Mean ± SEM gastric fluid pH, 2.76 ± 1.53 (range, 1.39-8.0), aspirated just prior to gastroscopic examination, was low and variable in horses in this study. There was no significant day or treatment effect on gastric juice pH during the study period. On the mornings of evaluation, the E-250 and E-SLH were not fed on the mornings until after the horses underwent gastroscopy.

### Blood gas values

**Figure 2:**
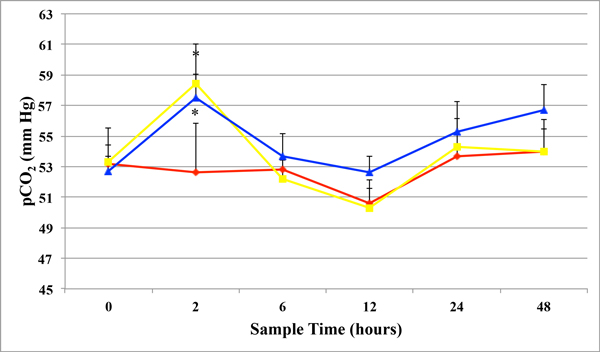
**Partial Pressure of CO_2_** Mean partial pressure of CO_2_ (pCO_2_) + SEM in whole heparinized venous blood in control, Egusin 250^®^, and Egusin SLH^®^ treated horses before treatment (0), 2, 6, 12, 24, and 48 hours after treatment. * denotes a significant (*P* < 0.05) difference from control values at the same time point. (controls = red line with diamonds; E-250 = yellow line with squares; E-SLH = blue line with triangles)

**Figure 3:**
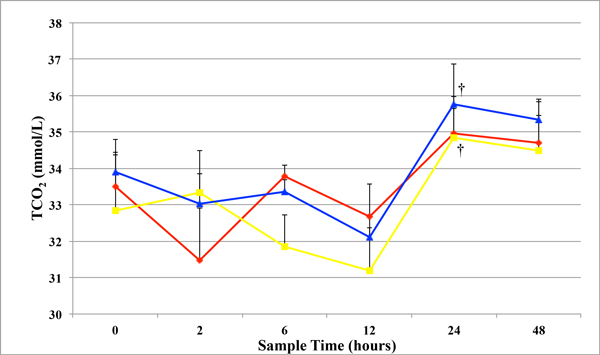
**Total CO_2_** Mean total CO_2_ (TCO_2_) + SEM in whole heparinized venous blood in control, Egusin 250^®^, and Egusin SLH^®^ treated horses before treatment (0), 2, 6, 12, 24, and 48 hours after treatment. (†) denotes a significant (*P* < 0.05) difference in measurement from the previous time within a group. (controls = red line with diamonds; E-250 = yellow line with squares; E-SLH = blue line with triangles)

[HCO_3_] did not significantly differ between days or within treatment groups for the study. However, mean pCO_2_ significantly increased 2 hours after initial treatment on day 0 in both E-250- and E-SLH-treated horses, when compared the untreated controls (Figure 2). Mean TCO_2_ was significantly higher in the E-250- and E-SLH-treated horses, 24 hours after treatment when compared to the 12-hour samples (Figure 3). However, mean TCO_2_ did not differ significantly from controls or between treatment groups throughout the rest of the treatment period (Figure 3).

## Discussion

The Egusin^®^ products, when mixed with sweet feed, were consumed by all horses. However, the E-250 (pellet formulation) was consumed more readily and completely than the E-SLH (powder formulation). The E-SLH sifted to the bottom of the feed tub and took longer for the horses to consume.

Two horses, an untreated control and E-250-treated horse, had signs of abdominal pain (colic) during the study. These horses were treated medically and recovered completely. Abdominal pain is a common clinical sign of gastric ulcers in horses and may have caused the signs seen in these two horses as they both had ulcers in the study. Although mineral oil and flunixin meglumine was administered to these two horses during the study, the authors feel that at the single dose administered, the drugs did not interfere with the results. No other clinical signs were noted in horses in this study.

Period one lasted for 28 days, while periods two and three were extended for a further 7 days after the feed-deprivation period (for a total of 35 days). This protocol change was based on a lack of response within the first 28 days, as well as previous studies with products containing pectin and lecithin and other ingredients that demonstrated improved ulcer scores after 30 days of treatment [18,20]. Thus, a 35-day, was incorporated into the later periods (2 and 3) to allow time to adequately evaluate the effects of treatment. In addition, statistical analysis focused on gastric ulcers scores in the 6 horses in each treatment group for these periods.

The intermittent feed-deprivation model used in this study was successful in worsening existing NG gastric ulcers, as mean EGUS scores significantly increased in the untreated controls and in the E-250-treated group after feed-deprivation. Egusin SLH^®^ treated horses did not show an increase in ulcer severity after feed-deprivation, and at day 28, there was no difference between control group and Egusin^®^ treatments. However, after 35 days of treatment, in control horses, gastric ulcer scores remained similar to day 28, while gastric ulcer scores decreased significantly in both Egusin^®^ -treated groups. Glandular ulcers were only seen in a few horses and there was not treatment effect.

In the study reported here, there was a decrease in mean EGUS gastric ulcer scores in the control horses after 21 days of treatment, but the scores were not significantly different from the other treatment groups at the same time point. Previous studies have shown that pasture-housed horses typically have less severe NG ulcers than stall-confined horses [30]. However, in a recent study, 71% of non-pregnant broodmares housed at pasture had gastric ulcers and some were severe [31]. The exact reason for the decrease in gastric ulcer severity in control horses in the study reported here is unknown, but might have been due to changes in management during this period. Typically, pasture grass in Louisiana has poor nutritional quality during the late fall and winter. The decreased feeding stress and increased nutrition could have led to the decreased severity of gastric ulcers in control horses during the first 21 days of the study compared to day 1. However, a similar decrease was not noted in the other treatment groups. Unfortunately, a feed analysis was not performed during the study period. During the study, horse body weight was measured before each gastroscopic examination and feed intake was adjusted to maintain body weight.

Egusin^®^-treated horses had improved NG gastric ulcer scores in this study after 35 days. The reason for the improvement in NG gastric ulcer scores might have been due to the ingredients found in the supplements. The Egusin**^®^** supplements used in this study contain surface-active and antacid ingredients, of which the amounts are proprietary. Surface-active ingredients include pectin (dried apple pectin pulp), soy lecithin, ß-glutan (micro-milled whole oats), whereas the antacids include dehydrated alfalfa meal, calcium carbonate, and sodium bicarbonate.

Pectin is a soluble complex polysaccharide derived from the cell wall of fruits and vegetables. Lecithin is a phospholipid derived from soybeans and is an emulsifying, lubricating agent that has surfactant properties. Pectin acts with lecithin to form a hydrophobic barrier on the gastric mucosal membranes, protecting them against the corrosive effects of gastric acids. In horses, the surface of the NG stomach is coated with dense osmophilic surface-active phospholipids (SAPLs), similar to the structure of pulmonary surfactant [32]. These hydrophobic SAPLs might provide the primary defense mechanism against exposure to hydrochloric and other organic acids found in gastric fluid. Feed additives containing pectin and lecithin have been previously evaluated in horses with gastric ulcers and the results have been mixed [19,20,33]. Egusin^®^ (which contains a proprietary mixture of pectin and lecithin) treatment significantly decreased NG gastric ulcer severity, when compared to untreated control horses, but there was not a treatment effect until day 35. In two previous studies, pectin and lecithin containing supplements decreased NG gastric ulcer severity after 10 days [33] and after 30 days [20] of treatment. Results in the later study, where horses were fed for 30 days, were similar to data in the study presented here, where it took 35 days to see a treatment effect in the Egusin^®^-treated horses.

However in another study, pectin and lecithin-treatment failed to prevent NG gastric ulcers from worsening after a 96-hour feed-deprivation period [19]. The results from that study were similar to the results from the study presented here, where Egusin 250^®^ treatment failed to prevent NG gastric ulcers from worsening during the feed-deprivation period[19]. However, E-SLH prevented the worsening of NG gastric ulcers during the period of feed-deprivation, but there was no treatment effect at day 28, when Egusin SLH^®^ was compared to the other treatment groups. Therefore the results should not be over interpreted as a response to treatment. It should be noted, however, that Egusin SLH^®^ has a higher concentration of pectin and lecithin and other ingredients than Egusin 250^®^, which could have accounted for this effect.

In addition to surface acting phospholipid compounds, Egusin^®^ products contain buffering agents, including calcium carbonate and sodium bicarbonate, which are commonly used used in people and horses to buffer stomach contents and neutralize gastric juice pH. No studies have critically evaluated the effects of antacids on gastric ulcer scores in horses. However, calcium carbonate reversed the damaging effects of hydrochloric acid on sodium and chloride transport across the NG mucosa in an *in vitro* Ussing chamber model [27,28]. In addition, one of the authors (FMA) showed that a supplement (Neigh-Lox®, Kentucky Performance Products, LLC, Versailles, KY) containing calcium carbonate fed to horses for 30 days increased gastric juice pH for 2 hours after feeding, but failed to improve gastric ulcer scores (unpublished data, Frank M. Andrews). This is similar to previous studies where antacids increase gastric juice pH for approximately 2 hours after administration [34]. Unfortunately, gastric juice pH was not measured two hours after feeding in the horses in the study. In addition to calcium carbonate, Egusin^®^ also contains sodium bicarbonate, which is not commonly used to treat gastric ulcers in horses and has not been critically evaluated for that purpose. However, it may increase gastric juice pH. In addition, sodium bicarbonate, when fed orally, can effect on blood pH. Sodium bicarbonate “milkshakes” have been generally speculated to help racing horses by increasing the pH of the blood, buffering lactic acid, and delaying onset of fatigue in muscles [35]. The generally accepted level of TCO_2_ in Thoroughbred racing in the United States is below 37 mmol/l [36]. Values that exceed 37 mmol/l are considered doping. The mean TCO_2_ values in the horses in this study did not exceed 37 mmol/l, despite the presence of calcium carbonate and sodium bicarbonate in the supplements. However, it is unlikely that the sodium bicarbonate concentration found in the Egusin^®^ products exceeds 0.5 g/kg, which has been shown to cause the blood TCO_2_ to increase above doping values.

The other ingredient that might have provided some buffering capacity is dried alfalfa meal. Although there are no studies evaluating the use of dried alfalfa meal in horses with gastric ulcers, horses fed alfalfa hay had higher gastric juice pH and lower NG gastric ulcer scores than the same horses fed bromegrass hay [37]. In another study, horses exercised and fed alfalfa hay had less severe NG gastric ulcers than horse that were fed grass hay [38]. It was hypothesized that the calcium carbonate in the alfalfa hay buffered stomach acid or directly inhibited gastric acid secretion as has been shown in rats fed diets containing 2% calcium carbonate [38]. It is unlikely that the concentration of calcium carbonate found in the dried alfalfa meal found in Egusin^®^ would be equivalent to that found in baled alfalfa hay, therefore its effect is likely minimal.

Gastric fluid pH was generally low and variable but not significantly different in Egusin^®^ treated horses when compared to untreated controls. Gastric fluid pH can be affected by stomach contents and the location in which the sample was taken [39]. A single sample aspirated from the stomach of horses can be variable and might not as accurate as continuous monitoring with a pH probe. However, gastric juice pH in the study reported here was similar to measurements where samples were collected via gastric cannulae and by continuous monitoring [18,37,40]. Neither Egusin^®^ product significantly altered gastric fluid pH at the times measured. However, perhaps a significant difference might have been seen if sampling would have been obtained within 2 hours of treatment, but may not have had a significant effect on gastric ulcer healing.

Other nutritional ingredients found in these Egusin^®^ products may have decreased NG gastric ulcer scores in these horses. ß-glucan has been shown to form gels in the stomach of other species and acts to coat the stomach lining. Studies in rats have shown antiulcergenic effects of ß-(1-->3)-glucosyl-linkage on beta-1-->3)-glucan itself is an important part of the active principle for anti-ulcerogenesis [41]. Studies on the effects of ß-glucan have not been reported in horses, but may be similar to those seen in rats. Also, the polar lipids in these supplements are a rich source of phospholipids and aid in re-establishing the stomach lining. However, there are no studies reported in horses on the effect of polar lipids on gastric ulcer scores in horses.

## Conclusions

The results of this study confirm that stall-confined horses undergoing feed-deprivation have a significant increase in the severity of NG gastric ulcers. However, it should be noted that a change in management from pasture to stall confinement and regular feeding could lower NG gastric ulcer scores in horses. Control horses had significantly decreased gastric ulcer scores after 21 days of stall-confinement, but these values were not different from the other treatment groups. Based on the data reported here, it appears that the ingredients contained in Egusin 250^®^ were not effective in reducing gastric ulcer severity during feed-deprivation, whereas the similar ingredients in Egusin SLH^®^, which are higher in concentration than Egusin 250^®^, may have aided in blunting an increase in NG gastric severity during feed-deprivation, although gastric ulcer scores increased in all treatment groups. Futhermore, gastric ulcer scores decreased in both Egusin^®^ -treated groups after 35 days of feeding (7 days after feed-deprivation). Thus, Egusin^®^ supplements fed twice daily for 35 days might reduce gastric ulcers scores after feed stress without exceeding threshold concentrations of bicarbonate.

## List of abbreviations

EGUS: equine gastric ulcer syndrome; E-250: Egusin 250^®^; E-SLH: Egusin SLH^®^; NG: nonglandular; [HOC_3_]: bicarbonate concentration; TCO_2_: total carbon dioxide; pCO_2_: partial pressure of carbon dioxide; SEM: standard error of the mean; SAPL: surface-active phospholipids

## Competing Interests

The study was funded by a grant from the Equine Health Studies Program, Equine Foundation, Louisiana State University School of Veterinary Medicine. The Egusin SLH^®^ and 250^®^ were supplied by the Centaur Corporation. The authors have no competing interest in the products tested.

## Authors’ Contributions

Drs. FMA and MCW contributed to study design. Drs. FMA, MCW, NKH and Mr. FG and Mr. MLK contributed to data collection and study execution. Data analysis was performed by Drs. MCW and MTK. Drs. MCW, MTK, and FMA contributed to data interpretation. Drs. MCW and FMA prepared the manuscript and Dr. NKH, Mr. FG and Mr. MLK contributed to editing and proofing reading of the manuscript.

## Declarations

This article has been published as part of *BMC Veterinary Research* Volume 10 Supplement 1, 2014: Selected articles from the Eleventh International Equine Colic Research Symposium. The full contents of the supplement are available online at http://www.biomedcentral.com/bmcvetres/supplements/10/S1. Publication of this supplement has been funded by The British Equine Veterinary Association.
